# Disparities for Asian American Medical Students in Alpha Omega Alpha and Gold Humanism Honor Societies

**DOI:** 10.1001/jamanetworkopen.2026.5168

**Published:** 2026-04-06

**Authors:** David H. Yang, Mytien Nguyen, Lindy Zhang, Jiun-Ruey Hu, Simona C. Kwon, Stella S. Yi, Lan N. Đoàn, David Henderson, Alexandra M. Hajduk, Sarwat I. Chaudhry, B. U. K. Li, Dowin Boatright

**Affiliations:** 1 Department of Emergency Medicine, Yale University School of Medicine, New Haven, Connecticut; 2 Yale University School of Medicine, New Haven, Connecticut; 3 Sidney Kimmel Comprehensive Cancer Center, Johns Hopkins University School of Medicine, Baltimore, Maryland; 4 Department of Oncology, Johns Hopkins University School of Medicine, Baltimore, Maryland; 5 Department of Cardiology, Smidt Heart Institute, Cedars-Sinai Medical Center, Los Angeles, California; 6 Department of Population Health, NYU Langone Health, New York, New York; 7 Department of Family Medicine, University of Connecticut School of Medicine, Farmington; 8 Department of Pediatrics, Medical College of Wisconsin, Milwaukee; 9 Department of Emergency Medicine, NYU Langone Health, New York, New York

## Abstract

**Importance:**

Membership in both the Alpha Omega Alpha (AOA) and Gold Humanism Honor Society (GHHS) is positively associated with career advancement. Prior studies have shown that Asian American medical students are less likely to be selected for these societies, but it is unknown whether representation among specific Asian American subgroups differ.

**Objective:**

To examine the association between AOA and GHHS membership and self-reported ethnicity among Asian American students at US doctor of medicine (MD)–granting medical schools.

**Design, Setting, and Participants:**

This retrospective cross-sectional study analyzed deidentified data from the Association of American Medical Colleges, focusing on allopathic medical students graduating between 2018 and 2021. Data analysis was conducted from July 10, 2024, to January 26, 2026.

**Exposure:**

Self-reported race and Asian ethnicity.

**Main Outcomes and Measures:**

The primary outcome was AOA and GHHS membership at graduation. Multivariable logistic regression was performed, adjusting for Medical College Admission Test score, childhood income, sex, and sexual orientation, and clustered by medical school.

**Results:**

Among 55 632 graduating medical students, 28 127 (50.6%) self-identified as female and 10 867 (19.5%) as Asian American. AOA membership was reported by 10 126 students (18.2%), and GHHS membership was reported by 8623 students (15.5%). Bangladeshi (odds ratio [OR], 0.35; 95% CI, 0.20-0.61), Chinese (OR, 0.51; 95% CI, 0.44-0.58), Filipino (adjusted OR, 0.44; 95% CI, 0.29-0.65), Indian (OR, 0.56; 95% CI, 0.50-0.63), Japanese (OR, 0.48; 95% CI, 0.28-0.81), Korean (OR, 0.41; 95% CI, 0.33-0.51), Pakistani (OR, 0.46; 95% CI, 0.34-0.63), Taiwanese (OR, 0.38; 95% CI, 0.28-0.51), and Vietnamese (OR, 0.56; 95% CI, 0.45-0.71) students were less likely to be AOA members than White students. Chinese (OR, 0.67; 95% CI, 0.58-0.78), Korean (OR, 0.55; 95% CI, 0.43-0.69), and Taiwanese (OR, 0.67; 95% CI, 0.49-0.91) students were less likely to be GHHS members compared with White students.

**Conclusions and Relevance:**

This cross-sectional study of graduating medical students found widespread underrepresentation of most Asian American subgroups in AOA membership and for Chinese, Korean, and Taiwanese medical students in GHHS membership. This underscores the importance of disaggregating Asian American individuals in medicine to unmask disparities and provide opportunities to promote greater inclusion in medicine.

## Key Points

**Question:**

How are Asian American subgroups represented in Alpha Omega Alpha (AOA) and Gold Humanism Honor Society (GHHS) membership?

**Findings:**

This cross-sectional study analyzed Association of American Medical Colleges data for 55 632 US medical school graduates between 2018 and 2021. Compared with White students, graduates self-identifying as Asian American were less likely to be AOA members, and Chinese, Korean, and Taiwanese graduates were less likely to be GHHS members.

**Meaning:**

These findings suggest there are disparities in AOA membership for most Asian American students and in GHHS membership for Chinese, Korean, and Taiwanese students that should inform residency selection and career advancement more broadly.

## Teaser

This cross-sectional study investigates whether there are representational disparities in medical student membership to Alpha Omega Alpha and Gold Humanism Honor Society among Asian American subgroups.

## Introduction

While Asian American students in aggregate represent over 20% of medical students overall, this population comprises over 40 different ethnic groups, each with unique cultural preferences, languages, and a variable level of representation within medical academia.^1^ Recent research using disaggregated data has identified that Cambodian, Filipino, Indonesian, and Laotian Americans are underrepresented in medical school relative to the general population.^2^ In addition, most Asian American subgroups shift toward underrepresentation with successive academic career stage, from residency to faculty to chairperson to dean levels of leadership.^3^ While recent studies have identified specific barriers that Asian American people face within their training, such as lack of competent mentorship and other racial stereotypes and discrimination,^2,4,5,6,7,8,9,10,11,12,13,14^ specific factors that contribute toward this shift in underrepresentation with successive academic career stages remain poorly understood.

The Gold Humanism Honor Society (GHHS) and Alpha Omega Alpha (AOA) are 2 medical student honor societies where membership can be an influential early factor for career advancement and residency selection.^15,16^ Membership to either society has been associated with future success in academic medicine, from being more likely to match into graduate medical education programs to achieving higher faculty ranks.^17,18,19^ GHHS uses student peer nomination, while AOA uses academic rank for membership eligibility.^20^ Both societies use a deliberative body to finalize membership.^21,22^ A recent study found that Asian American medical graduates are less likely to obtain AOA or GHHS membership.^23^ This difference was thought to be related to disparities in clinical evaluations for AOA membership and biases against Asian American students by members of the deliberative bodies.^21,22,24^ That study was limited in its aggregation of Asian American into a single racial and ethnic group, despite evidence that aggregation obscures inequities in health care experiences.^2,12^

Further investigation into this disparity in honor society membership could help us to understand whether some Asian American subgroups are progressing differently early in their careers, informing strategies to achieve equitable leadership representation. The objective of our study was to examine the association of GHHS and AOA membership with disaggregated Asian American subgroup. We hypothesized that there would be variability in honor society membership with disaggregation of Asian American subgroups.

## Methods

### Study Design and Population

This cross-sectional study used deidentified data from the Association of American Medical Colleges (AAMC),^25^ focusing on medical students graduating between 2018 and 2021 from schools offering both AOA and GHHS chapters. We used data supplied by AAMC from the American Medical College Application Service, the Graduation Questionnaire (GQ), and the Electronic Residency Application Service. We excluded students who attended medical schools designated as historically Black colleges and universities or were in Puerto Rico because of a greater proportion of underrepresented medical students.^26,27^ We excluded students who attended medical schools without GHHS and AOA chapters. Data analysis was conducted from July 10, 2024, to January 26, 2026. This study was deemed as exempt and approved by the Yale University institutional review board and followed the Strengthening the Reporting of Observational Studies in Epidemiology (STROBE) reporting guidelines for cross-sectional studies.

### Variables

The primary outcome was self-reported GHHS and AOA membership status. The primary exposure was self-reported race and ethnicity. Self-reported race and ethnicity was specified by the AAMC and categorized as American Indian or Alaska Native; Asian (hereafter Asian American); Black or African American; Hispanic, Latino, or of Spanish origin (hereafter Hispanic or Latino); multiracial; Native Hawaiian or Pacific Islander; non-Hispanic White; or other race or ethnicity.^28^ Asian American students were disaggregated by available self-reported Asian ethnicity (Asian Other, Bangladeshi, Cambodian, Chinese, Filipino, Indian, Indonesian, Japanese, Korean, Laotian, Pakistani, Taiwanese, and Vietnamese). We combined Cambodian, Laotian, and Indonesian into Other Southeast Asian group because of small sample sizes and because they are numerically underrepresented among medical students compared with the US population.^2,29^ Students who identified as Asian American and did not identify as another race and ethnicity were grouped into Asian Other if they did not report an Asian ethnicity or identified with multiple Asian ethnicities (ie, Filipino and Taiwanese). Students were grouped into multiracial if they selected multiple races and ethnicities (ie, non-Hispanic White and Asian American).

Other demographic variables included sex (male vs female), sexual orientation (heterosexual or straight, bisexual, gay or lesbian), and childhood family annual income. Childhood family income was defined as either low income or not low income, with a low-income cutoff of $50 000, or approximately 200% of the federal poverty level for a family of 4.^30^ The Medical College Admission Test (MCAT) score quintile was included as a potential confounding variable, with quintiles for integration of the MCAT that preceded April 2015 and the current MCAT.^31^

### Statistical Analysis

We used simple descriptive characteristics to summarize sample characteristics by honor society membership. Multiple imputation was performed using a fully conditional specification, with each incomplete variable iteratively modeled using a separate model conditional on other variables in the dataset, to impute all missing data.^32,33^ Specifically, missing values in binary variables were imputed using logistic regression and missing values in other categorical variables were imputed using proportional odds model. We included all demographic variables and the MCAT score in the imputation model and created 20 imputed datasets using the R package mice. Analyses were conducted for each imputed dataset, and the results of all datasets were summarized. We used a logistic regression model to examine the association between honor society membership and race and ethnicity, adjusting for sex, sexual orientation, childhood income, and MCAT score, and clustering by medical school. We used a second model controlling for the same variables with Asian American in aggregate. Clustering was considered by estimating for random effects. As the sample characteristics (eTable 1 in Supplement 1) and results were similar with and without imputation, only results from multiple imputation are presented. We performed a sensitivity analysis where we compared the association of honor society membership when the Other Southeast Asian subgroup was in aggregate compared with when it was disaggregated. We found that aggregation of this group did not change results; therefore, we opted to report results in aggregate due to small sample size (eTable 4 and eTable 5 in Supplement 1). Analyses were performed with R statistical software version 4.2.0 (R Project for Statistical Computing), and *P* < .05 was set as the threshold for statistical significance.

## Results

A total of 63 800 responses were collected between 2018 and 2021, with 8168 excluded responses (Figure). Our final cohort included 55 632 students, of whom 28 127 (50.6%) self-identified as female (Table 1), 10 867 (19.5%) as Asian American, 1876 (3.4%) as bisexual, and 2043 (3.7%) as lesbian or gay, and 8046 (14.5%) as students with childhood annual family income less than $50 000. AOA membership was reported by 10 126 students (18.2%) and GHHS membership was reported by 8623 students (15.5%).

**Figure.**
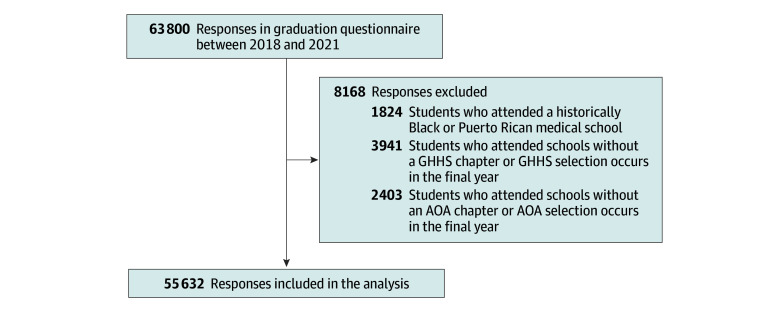
Flow Diagram for Graduating Medical Students Eligible and Excluded From the Study AOA indicates Alpha Omega Alpha; GHHS, Gold Humanism Honor Society.

**Table 1.** Demographic Characteristics of Graduating Medical Students by AOA and GHHS Membership, 2018 to 2021

Characteristic	Total No. (N = 55 632)	Participants, No. (%)					
		GHHS member			AOA member		
		No (n = 47 009 [84.5%])	Yes (n = 8623 [15.5%])	*P* value	No (n = 45 506 [81.8%])	Yes (n = 10 126 [18.2%])	*P* value
Race and ethnicity							
American Indian or Alaska Native	122	107 (87.7)	15 (12.3)	<.001	105 (86.1)	17 (13.9)	<.001
Asian American	10 867	9396 (86.4)	1471 (13.5)		9364 (86.2)	1503 (13.8)	
Asian Other^a^	1546	1303 (84.3)	243 (15.7)		1323 (85.6)	223 (14.4)	
Bangladeshi	165	145 (87.9)	20 (12.1)		150 (90.9)	15 (9.1)	
Chinese	2270	2016 (88.8)	254 (11.2)		1943 (85.6)	327 (14.4)	
Filipino	293	257 (87.7)	36 (12.3)		260 (88.7)	33 (11.3)	
Indian	3513	2950 (84.0)	563 (16.0)		2960 (84.3)	553 (15.7)	
Japanese	167	141 (84.4)	26 (15.6)		144 (86.2)	23 (13.8)	
Korean	1045	950 (90.9)	95 (9.1)		940 (90.0)	105 (10.0)	
Other Southeast Asian^b^	50	48 (96.0)	2 (4.0)		47 (94.0)	3 (6.0)	
Pakistani	484	418 (86.4)	66 (13.6)		430 (88.8)	54 (11.2)	
Taiwanese	570	507 (88.9)	63 (11.1)		501 (87.9)	69 (12.1)	
Vietnamese	764	661 (86.5)	103 (13.5)		666 (87.2)	98 (12.8)	
Black or African American	2808	2197 (78.2)	611 (21.8)		2644 (94.2)	164 (5.8)	
Hispanic, Latino, or of Spanish origin	4479	3773 (84.2)	706 (15.8)		3983 (88.9)	496 (11.1)	
Multiracial	1769	1495 (84.5)	274 (15.5)		1453 (82.1)	316 (17.9)	
Native Hawaiian or Pacific Islander	47	40 (85.1)	7 (14.9)		44 (93.6)	3 (6.4)	
Non-Hispanic White	31 638	26 686 (84.3)	4952 (15.7)		24 589 (77.7)	7049 (22.3)	
Other^c^	1098	900 (82.0)	198 (18.0)		923 (84.1)	175 (15.9)	
Missing	2804	2415 (86.1)	289 (10.3)		2401 (85.6)	403 (14.4)	
Sex							
Female	28 127	22 930 (81.5)	5197 (18.5)	<.001	22 935 (81.5)	5192 (18.5)	<.001
Male	27 505	24 079 (87.5)	3426 (12.5)		22 571 (82.1)	4934 (17.9)	
Sexual orientation							
Bisexual	1876	1516 (80.8)	360 (19.2)	<.001	1605 (85.6)	271 (14.4)	<.001
Heterosexual or straight	47 480	40 129 (84.5)	7351 (15.5)		38 539 (81.2)	8941 (18.8)	
Gay or lesbian	2043	1670 (81.7)	373 (18.3)		1670 (81.7)	373 (18.3)	
Missing	4233	3694 (87.3)	539 (12.7)		3692 (87.2)	541 (12.8)	
Childhood family annual income							
Low (<$50 000)	8046	6486 (85.1)	1200 (14.9)	<.001	7156 (88.9)	890 (11.1)	<.001
Not low (≥$50 000)	34 344	28 845 (84.0)	5499 (16.0)		27 346 (79.6)	6998 (20.4)	
Missing	13 242	11 318 (85.5)	1924 (14.5)		11 004 (83.1)	2238 (16.9)	
MCAT quintile							
First	10 274	8522 (82.9)	1752 (17.1)	<.001	9299 (90.5)	975 (9.5)	<.001
Second	15 037	12 688 (84.4)	2349 (15.6)		12 629 (84.0)	2408 (16.0)	
Third	12 314	10 376 (84.3)	1938 (15.7)		9791 (79.5)	2523 (20.5)	
Fourth	8831	7532 (85.3)	1299 (14.7)		6780 (76.8)	2051 (23.2)	
Fifth	7534	6537 (86.8)	997 (13.2)		5668 (75.2)	1866 (24.8)	
Missing	1642	1354 (82.5)	288 (17.5)		1339 (81.5)	303 (18.5)	

Abbreviations: AOA, Alpha Omega Alpha; GHHS, Gold Humanism Honor Society; MCAT, Medical College Admissio Test; NA, not applicable.

^a^ Asian Other includes (1) those who identified as Asian but did not provide additional ethnicity and (2) those who identified as multiple Asian ethnicities.

^b^ Other Southeast Asian includes Cambodian, Indonesian, and Laotian.

^c^ Other includes those who responded as other, unknown, or declined to respond to the Graduation Questionnaire.

### AOA Membership

In total, 10 126 medical students (18.2%) were selected as AOA members. In our cohort, 7049 of 31 638 White students (22.3%) were AOA members, compared with 1503 of 10 867 Asian American students (13.8%) (Table 1). In the disaggregated Asian cohort, 15 of 165 Bangladeshi (9.1%), 327 of 2270 Chinese (14.4%), 33 of 293 Filipino (11.3%), 553 of 3513 Indian (15.7%), 23 of 167 Japanese (13.8%), 105 of 1045 Korean (10.0%), 3 of 50 Other Southeast Asian (6.0%), 54 of 484 Pakistani (11.2%), 69 of 570 Taiwanese (12.1%), and 98 of 764 Vietnamese American (12.8%) students were AOA members.

In the fully adjusted model, Asian American medical students were less likely to be AOA members compared with White students (OR, 0.51; 95% CI, 0.48-0.55) (eTable 2 and eTable 6 in Supplement 1). Most Asian American subgroups (10 of 11) were less likely to be AOA members compared with White students, including Asian Other (OR, 0.55; 95% CI, 0.46-0.67), Bangladeshi (OR, 0.35; 95% CI, 0.20-0.61), Chinese (OR, 0.51; 95% CI, 0.44-0.58), Filipino (OR, 0.44; 95% CI, 0.29-0.65), Indian (OR, 0.56; 95% CI, 0.50-0.63), Japanese (OR, 0.48; 95% CI, 0.28-0.81), Korean (OR, 0.41; 95% CI, 0.33-0.51), Pakistani (OR, 0.46; 95% CI, 0.34-0.63), Taiwanese (OR, 0.38; 95% CI, 0.28-0.51), and Vietnamese (OR, 0.56; 95% CI, 0.45-0.71) students (Table 2). There was no significant difference in AOA membership between Other Southeast Asian students and White students (OR, 0.32; 95% CI, 0.10-1.05).

**Table 2.** ORs of Membership in Alpha Omega Alpha Honor Society by Student Demographic Characteristic

Characteristic	OR (95% CI)	
	Unadjusted model^a^	Adjusted model^b^
Race and ethnicity		
American Indian or Alaska Native	0.58 (0.35-0.96)	0.84 (0.47-1.49)
Asian American^c^	0.53 (0.50-0.57)	0.51 (0.48-0.55)
Asian Other^d^	0.55 (0.48-0.64)	0.55 (0.46-0.67)
Bangladeshi	0.32 (0.19-0.55)	0.35 (0.20-0.61)
Chinese	0.56 (0.49-0.63)	0.51 (0.44-0.58)
Filipino	0.44 (0.31-0.63)	0.44 (0.29-0.65)
Indian	0.62 (0.56-0.68)	0.56 (0.50-0.63)
Japanese	0.54 (0.34-0.84)	0.48 (0.28-0.81)
Korean	0.37 (0.30-0.46)	0.41 (0.33-0.51)
Other Southeast Asian (Cambodian, Indonesian, Laotian)	0.20 (0.06-0.63)	0.32 (0.10-1.05)
Pakistani	0.42 (0.35-0.59)	0.46 (0.34-0.63)
Taiwanese	0.46 (0.35-0.59)	0.38 (0.28-0.51)
Vietnamese	0.50 (0.40-0.62)	0.56 (0.45-0.71)
Black or African American	0.21 (0.18-0.25)	0.38 (0.31-0.45)
Hispanic, Latino, or of Spanish origin	0.42 (0.38-0.46)	0.54 (0.48-0.61)
Multiracial	0.75 (0.66-0.86)	0.76 (0.66-0.88)
Native Hawaiian or Pacific Islander	0.23 (0.07-0.74)	0.29 (0.07-1.24)
Non-Hispanic White	1 [Reference]	1 [Reference]
Other^e^	0.64 (0.54-0.76)	0.76 (0.63-0.92)
Sex		
Female	1 [Reference]	1 [Reference]
Male	0.97 (0.93-1.01)	0.81 (0.77-0.86)
Sexual orientation		
Bisexual	0.72 (0.63-0.83)	0.66 (0.56-0.76)
Heterosexual or straight	1 [Reference]	1 [Reference]
Gay or lesbian	0.96 (0.86-1.08)	0.96 (0.85-1.10)
Childhood family annual income		
Low (<$50 000)	1 [Reference]	1 [Reference]
Not low (≥$50 000)	2.08 (1.93-2.24)	1.54 (1.42-1.66)
MCAT quintile		
First	NA	1 [Reference]
Second	NA	1.73 (1.58-1.90)
Third	NA	2.41 (2.19-2.65)
Fourth	NA	3.02 (2.72-3.35)
Fifth	NA	3.63 (3.24-4.06)

Abbreviations: NA, not applicable; OR, odds ratio.

^a^ Adjusted for clustering by school.

^b^ Adjusted for clustering by school and for demographic variables and MCAT quintile.

^c^ ORs for Asian Americans in aggregate were calculated as a separate model reported in eTable 1 in Supplement 1.

^d^ Asian Other includes (1) those who identified as Asian but did not provide additional ethnicity and (2) those who identified as multiple Asian ethnicities.

^e^ Other includes those who responded as other, unknown, or declined to respond to the Graduation Questionnaire.

### GHHS Membership

In total, 8623 medical students (15.5%) were selected as GHHS members. In our cohort, 4952 White students (15.7%) were GHHS members, compared with 1471 Asian American students (13.5%) (Table 1). In the disaggregated Asian cohort, 20 Bangladeshi (12.1%), 254 Chinese (11.2%), 36 Filipino (12.3%), 563 Indian (16.0%), 26 Japanese (15.6%), 95 Korean (9.1%), 2 Other Southeast Asian (4.0%), 66 Pakistani (13.6%). 63 Taiwanese (11.1%), and 103 Vietnamese students (13.5%) were GHHS members.

In the fully adjusted model, Asian American medical students were less likely to be GHHS members compared with White students (OR, 0.84; 95% CI, 0.78-0.90) (eTable 3 and eTable 7 in Supplement 1). Only 3 of 11 Asian American subgroups were less likely to be GHHS members compared with White students, including Chinese (OR, 0.67; 95% CI,0.58-0.78), Korean (OR 0.55; 95% CI, 0.43-0.69), and Taiwanese (OR, 0.67; 95% CI, 0.49-0.91) students (Table 3). Other Asian American subgroups (Bangladeshi, Filipino, Indian, Japanese, Other Southeast Asian, Pakistani, and Vietnamese) were as likely to be GHHS members compared with White students (eTables 2, 3, 6, and 7 in Supplement 1).

**Table 3.** ORs of Membership in Gold Humanism Honor Society by Student Demographic Characteristic

Characteristic	OR (95% CI)	
	Unadjusted model^a^	Adjusted model^b^
Race and ethnicity		
American Indian or Alaska Native	0.74 (0.43-1.27)	0.84 (0.47-1.48)
Asian American^c^	0.83 (0.78-0.89)	0.84 (0.78-0.90)
Asian Other^d^	0.99 (0.85-1.14)	1.01 (0.83-1.21)
Bangladeshi	0.72 (0.45-1.15)	0.69 (0.41-1.15)
Chinese	0.65 (0.57-0.75)	0.67 (0.58-0.78)
Filipino	0.77 (0.55-1.10)	0.70 (0.47-1.04)
Indian	1.03 (0.93-1.13)	1.05 (0.94-1.16)
Japanese	0.99 (0.64-1.51)	0.66 (0.38-1.15)
Korean	0.55 (0.45-0.68)	0.55 (0.43-0.69)
Other Southeast Asian (Cambodian, Indonesian, Laotian)	0.24 (0.06-0.93)	0.30 (0.07-1.24)
Pakistani	0.84 (0.65-1.09)	0.84 (0.63-1.13)
Taiwanese	0.65 (0.50-0.85)	0.67 (0.49-0.91)
Vietnamese	0.82 (0.66-1.01)	0.84 (0.67-1.05)
Black or African American	1.50 (1.36-1.65)	1.39 (1.24-1.56)
Hispanic, Latino, or of Spanish origin	0.99 (0.91-1.08)	0.99 (0.89-1.09)
Multiracial	0.98 (0.86-1.12)	0.93 (0.80-1.08)
Native Hawaiian or Pacific Islander	0.91 (0.41-2.02)	0.99 (0.38-2.61)
Non-Hispanic White	1 [Reference]	1 [Reference]
Other^e^	1.20 (1.02-1.41)	1.34 (1.12-1.60)
Sex		
Female	1 [Reference]	1 [Reference]
Male	0.63 (0.60-0.66)	0.65 (0.61-0.69)
Sexual orientation		
Bisexual	1.28 (1.14-1.45)	1.16 (1.01-1.32)
Heterosexual or straight	1 [Reference]	1 [Reference]
Gay or lesbian	1.19 (1.06-1.34)	1.34 (1.18-1.53)
Childhood family annual income		
Low (<$50 000)	1 [Reference]	1 [Reference]
Not low (≥$50 000)	1.09 (1.02-1.17)	1.12 (1.04-1.20)
MCAT quintile		
First	NA	1 [Reference]
Second	NA	0.95 (0.88-1.03)
Third	NA	0.95 (0.87-1.04)
Fourth	NA	0.90 (0.81-0.99)
Fifth	NA	0.82 (0.74-0.92)

Abbreviations: NA, not applicable; OR, odds ratio.

^a^ Adjusted for clustering by school.

^b^ Adjusted for clustering by school and for demographic variables and MCAT quintile.

^c^ ORs for Asian Americans in aggregate were calculated as a separate model reported in eTable 2 in Supplement 1.

^d^ Asian Other includes (1) those who identified as Asian but did not provide additional ethnicity and (2) those who identified as multiple Asian ethnicities.

^e^ Other includes those who responded as other, unknown, or declined to respond to the Graduation Questionnaire.

## Discussion

This national cross-sectional study of graduating US MD-granting medical students highlights widespread underrepresentation of most Asian American subgroups in the membership within the prestigious AOA and GHHS medical student honor societies. First, students from 10 of 11 Asian American subgroups were less likely than White students to be AOA members, with 6 groups less than half as likely to be AOA members. Second, in GHHS membership, disparities were identified for Chinese, Korean, and Taiwanese American medical students, but the 8 other Asian American subgroups experienced equitable representation.

Our findings add to the growing evidence base that there are persistent disparities in AOA membership, with almost all Asian American subgroups underrepresented. Disparities in AOA membership were first characterized in 2017, where Boatright et al^24^ reported that Asian American students were half as likely to be AOA members compared with their White counterparts. The magnitude of this disparity for Asian American subgroups is noteworthy, with Bangladeshi, Filipino, Japanese, Korean, Pakistani, and Taiwanese students less than half as likely to be AOA members compared with their White classmates. This finding underscores the diverse experiences within the Asian American medical student population. In addition, Black, Hispanic or Latino, and multiracial students are less likely to be AOA members compared with White students nearly a decade after Boatright et al^24^ in 2017. The persistence of these disparities in AOA membership may represent a persistent vulnerability in the assessment of medical students with profound implications for the future physician workforce given the benefits of AOA membership on future opportunities in academic medicine.^17,18,19^ Acknowledging the persistent racial disparity in AOA membership and how this could limit opportunities for minority candidates, program directors and academic medicine leaders may want to pause and consider the use of AOA membership status in their method of candidate selection until this disparity is addressed on a national level. The national AOA society may consider refining their metrics for selecting members to provide transparency while mitigating the potential for bias and providing annual report on member demographics like race and ethnicity.^24^

Historically, AOA uses academic rank, which may include standardized test scores and clinical evaluations, as member eligibility.^20^ While we control for MCAT score in this study, additional research is warranted to examine the role of other standardized tests, like US Medical Licensing Examination scores, academic school ranking, and clinical evaluations on gaining AOA membership. A large body of evidence suggests that racial bias is present in clinical evaluations and written clerkship evaluations of medical students.^4,34,35^ Ross et al^34^ found that Asian American medical students were less likely to be described with standout words on their Medical Student Performance Evaluations, suggesting that racial stereotypes may contribute to how Asian American medical students are perceived and described. For example, the model minority myth, a racial stereotype that portrays Asian American individuals as academically successful, hardworking, and homogeneous, may create a façade of apparent privilege that masks the many challenges Asian American medical students face during their medical training, contributing to unrecognized academic struggles, worse academic scores and clinical evaluations, and a widened honor society membership gap.^36^ This form of racial stereotyping may also lead to inferior clerkship evaluations in other ways. In Zhang’s^13^ work on microaggressions toward Asian American medical students, a common theme was the presumption by faculty members that the Asian American student was quiet, which was further misinterpreted as lack of self-confidence or disengagement. In turn, this may contribute to inferior clinical evaluations, impacting their likelihood of honor society membership.^37^

We also found that significant disparities in GHHS membership exist for Asian American medical students. Prior research found that Asian American students were 20% less likely to be GHHS members compared with their White counterparts.^23^ In our analyses disaggregating Asian race, we found that this disparity exists for Chinese, Korean, and Taiwanese American medical students. As GHHS uses peer nomination, stereotyping and bias may contribute to the underrepresentation of Chinese, Korean, and Taiwanese medical students.^12^ The inequitable membership may stem from racial stereotyping from their peers,^38^ where some studies have shown that Asian American medical students report feeling invisible in the training environment and that they must work harder than their peers to be seen.^4,12^ Asian American medical students, especially Asian American women, feel that they are seen as worker bees and quiet and submissive in describing perceived barriers to leadership.^8^ As additional evidence, the second most prevalent source of the microaggressions experienced by Asian American medical students came from peer medical students.^13^ While GHHS can be lauded for equitable representation across other minority groups (American Indian or Alaska Native, Black or African American, Hispanic or Latino, and Native Hawaiian or Pacific Islander students),^23^ our findings highlight the importance of continued efforts to minimize biases in entrance to these societies. Additional inquiry should explore reasons for why these 3 Asian American subgroups are underrepresented in GHHS while other Asian American subgroups and other minoritized groups experience equitable representation. Specific to the GHHS peer nomination process, further studies should examine whether these 3 subgroups are less likely to be nominated by their peers as potential candidates or if the underrepresentation emerges afterwards with the deliberative body. Since deliberative bodies are present in the AOA and GHHS process, qualitative research focused on deliberative body members may help us understand how bias contributes to underrepresentation in each respective society.

Our findings add to the growing body of literature that supports disaggregation of Asian American individuals in medicine and the importance of disaggregation of medical workforce data, with implications for leaders of honor societies, residency programs, and other professional organizations. Prior research^2,3^ found that more Asian American subgroups are underrepresented among physicians along the academic career trajectory: 2 of 12 Asian American subgroups were underrepresented among medical students while 10 of 12 Asian American subgroups were underrepresented among residents and faculty. As honor society membership is associated with being more likely to match into residency, fellowship, and higher faculty rank, our finding that particular Asian American subgroups are underrepresented in honor society membership identifies one likely reason for the progressively diminished representation of Asian American individuals as residents and among academic faculty.^17,18,19^ This deepens our understanding of the bamboo ceiling,^7^ a phenomenon describing the underrepresentation of Asian American individuals in academic leadership and the barriers they face in career advancement, with disparity in membership likely contributing to a cascade that work to limit representation in medical leadership positions.^39^ Disaggregating workforce data unmasks disparities and provides opportunities to tailor mentorship and outreach to promote inclusion in medicine. Tailored efforts may counteract the model minority myth, the bamboo ceiling, and other barriers that different Asian American subgroups face, arresting the ascending cascade of biases that lead to underrepresentation of Asian American individuals in leadership.

### Limitations

This study has limitations. First, this study spanned graduating medical students between 2018 and 2021. Recent developments, from the rise of anti-Asian violence against health care workers to the recent US Supreme Court ruling against affirmative action in *Students for Fair Admissions (SFFA) v Harvard *and* SFFA v University of North Carolina *[600 US 181 2023], may have altered the experience of Asian American medical students.^12,40,41^ Second, subgroups used to disaggregate Asian Americans are defined by the GQ, and we define them as ethnicities to remain consistent with the GQ. However, these subgroups correspond to nationalities, each of which include several different ethnicities.^28^ For example, there are likely several distinct ethnicities among students who self-identify as Chinese, such as Han or Miao.^42^ The available data did not include many potential subgroups, including Burmese, Hmong, Nepalese, and other relatively small Asian American subgroups. Additionally, these data do not distinguish based on citizenship or immigration status. Third, there was relatively low representation of Cambodian, Indonesian, Laotian, Native Hawaiian or Pacific Islander, and American Indian or Alaska Native students, and this potentially contributed to the lack of statistically significant results for these groups. However, to our knowledge, this study is the first to provide disaggregated information on honor society membership at the medical school level. Fourth, our study only included students studying at MD-granting medical schools and osteopathic medical schools were not included. Future inquiry is needed to further examine this disparity in honor society membership and the role of racial stereotypes, including representation in other honor societies like the osteopathic honor society Sigma Sigma Phi. Fifth, while we controlled for MCAT, we could not control for potentially influential variables like US Medical Licensing Examination score, class rank, or clinical evaluations. Additional research should investigate the impact of these variables and explore the impact of biases on membership in both honor societies. Sixth, this study assesses racial and ethnic disparities within medical schools that induct members into honor societies prior to completion of the AAMC graduation questionnaire. Since this disparity was originally reported in 2017, some medical schools have suspended their affiliation with AOA or do not select students for AOA until after the residency match.^43,44^

## Conclusions

In this cross-sectional study of US MD-granting medical students, disaggregation of Asian American medical students demonstrated underrepresentation in both AOA and GHHS for Chinese, Korean, and Taiwanese medical students. Medical students in almost all Asian American subgroups were underrepresented in AOA. The findings of this study suggest that the diminished representation that Asian American individuals experience in medicine starts in their undergraduate medical education and warrant additional research to further examine this issue.

## Notes

Supplement 1. eTable 1. Sample demographics with and without multiple imputation eTable 2. Odds ratios of membership in Alpha Omega Alpha Honor Society by student demographic characteristic with aggregated race/ethnicity eTable 3. Odds ratios of membership in Gold Humanism Honor Society by student demographic characteristic with aggregated race/ethnicity eTable 4. Odds ratios of membership in Alpha Omega Alpha Honor Society by student demographic characteristic with further disaggregated data eTable 5. Odds ratio of membership in Gold Humanism Honor Society by student demographic characteristic with further disaggreated data eTable 6. Odds ratios of membership in Alpha Omega Alpha Honor Society by student demographic characteristic, not controlling for medical college admission test eTable 7. Odds ratios of membership in Gold Humanism Honor Society by student demographic characteristic, not controlling for medical college admission test

Supplement 2. Data Sharing Statement
