# Supplementary material for: Disparities for Asian American Medical Students in Alpha Omega Alpha and Gold Humanism Honor Societies
Source: JAMA Netw Open. 2026 Apr 6;9(4):e265168. doi: 10.1001/jamanetworkopen.2026.5168 (PMC13054621; doi:10.1001/jamanetworkopen.2026.5168)
Supplement: Supplement 1. — eTable 1. Sample demographics with and without multiple imputation eTable 2. Odds ratios of membership in Alpha Omega Alpha Honor Society by student demographic characteristic with aggregated race/ethnicity eTable 3. Odds ratios of membership in Gold Humanism Honor Society by student demographic characteristic with aggregated race/ethnicity eTable 4. Odds ratios of membership in Alpha Omega Alpha Honor Society by student demographic characteristic with further disaggregated data eTable 5. Odds ratio of membership in Gold Humanism Honor Society by student demographic characteristic with further disaggreated data eTable 6. Odds ratios of membership in Alpha Omega Alpha Honor Society by student demographic characteristic, not controlling for medical college admission test eTable 7. Odds ratios of membership in Gold Humanism Honor Society by student demographic characteristic, not controlling for medical college admission test [file jamanetwopen-e265168-s001.pdf]

Supplemental Online Content

Yang D, Nguyen M, Zhang L, et al. Disparities for Asian American medical students in Alpha Omega Alpha honor society and Gold Humanism honor society. *JAMA Netw Open.* 2026;9(4):e265168. doi:10.1001/jamanetworkopen.2026.5168

eTable 1. Sample demographics with and without multiple imputation

eTable 2. Odds ratios of membership in Alpha Omega Alpha Honor Society by student demographic characteristic with aggregated race/ethnicity

eTable 3. Odds ratios of membership in Gold Humanism Honor Society by student demographic characteristic with aggregated race/ethnicity

eTable 4. Odds ratios of membership in Alpha Omega Alpha Honor Society by student demographic characteristic with further disaggregated data

eTable 5. Odds ratio of membership in Gold Humanism Honor Society by student demographic characteristic with further disaggreated data

eTable 6. Odds ratios of membership in Alpha Omega Alpha Honor Society by student demographic characteristic, not controlling for medical college admission test

eTable 7. Odds ratios of membership in Gold Humanism Honor Society by student demographic characteristic, not controlling for medical college admission test

This supplemental material has been provided by the authors to give readers additional information about their work.

© 2026 Yang D et al. *JAMA Network Open*.

eTable1. Sample Demographics with and without multiple imputation

	Sample with Imputation (N=55632)	Sample without Imputation (N=37077)
**Race and Ethnicity**		
American Indian or Alaska Native	122 (0.2%)	90 (0.2%)
Asian American		
Asian Other[a]	1546 (2.8%)	797 (2.1%)
Bangladeshi	165 (0.3%)	127 (0.3%)
Cambodian	21 (0.0%)	16 (0.0%)
Chinese	2270 (4.1%)	1621 (4.4%)
Filipino	293 (0.5%)	212 (0.6%)
Indian	3513 (6.3%)	2425 (6.5%)
Indonesian	23 (0.0%)	14 (0.0%)
Japanese	167 (0.3%)	118 (0.3%)
Korean	1045 (1.9%)	732 (2.0%)
Laotian	6 (0.0%)	4 (0.0%)
Pakistani	484 (0.9%)	358 (1.0%)
Taiwanese	570 (1.0%)	377 (1.0%)
Vietnamese	764 (1.4%)	602 (1.6%)
Black or African American	2808 (5.0%)	1892 (5.1%)
Hispanic, Latino, or of Spanish Origin	4479 (8.1%)	3104 (8.4%)
Multiracial	1769 (3.2%)	1233 (3.3%)
Native Hawaiian or Pacific Islander	47 (0.1%)	29 (0.1%)
Non-Hispanic White	31638 (56.9%)	22610 (61.0%)
Other[b]	1098 (2.0%)	716 (1.9%)
**Sex**		
Female	28127 (50.6%)	19018 (51.3%)
Male	27505 (49.4%)	18059 (48.7%)
**Sexual Orientation**		
Bisexual	1876 (3.4%)	1395 (3.8%)
Heterosexual or Straight	47480 (85.3%)	34177 (92.2%)
Gay or Lesbian	2043 (3.7%)	1505 (4.1%)
**Childhood Income**		
Low Income (<$50,000)	8046 (14.5%)	6783 (18.3%)
Not Low Income (≥$50,000)	34344 (61.7%)	30294 (81.7%)
**MCAT Quintile**		
1st Quintile	10274 (18.5%)	7151 (19.3%)
2nd Quintile	15037 (27.0%)	10451 (28.2%)
3rd Quintile	12314 (22.1%)	8497 (22.9%)
4th Quintile	8831 (15.9%)	5982 (16.1%)
5th Quintile	7534 (13.5%)	4996 (13.5%)

© 2026 Yang D et al. *JAMA Network Open*.

[a] ”Asian Other” includes 1) those who identified as Asian but did not provide additional ethnicity and 2) those who identified as multiple Asian ethnicities.
[b] “Other” includes those who responded as Other, Unknown, or Declined to Respond to the Graduation Questionnaire.

eTable2. Odds ratios of membership in Alpha Omega Alpha Honor Society by student demographic characteristic with aggregated race/ethnicity

	**Odds ratio (95% CI)**		
**Characteristic**	(unadjusted) Model[a]	(adjusted) Model[b]	(adjusted) Model[c]
**Race and Ethnicity**			
American Indian or Alaska Native	0.58 (0.35,0.96)	0.61 (0.34,1.08)	0.84 (0.47,1.49)
Asian American	0.53 (0.50,0.57)	0.56 (0.52,0.60)	0.51 (0.48,0.55)
Black or African American	0.21 (0.18,0.25)	0.25 (0.21,0.30)	0.37 (0.31,0.45)
Hispanic, Latino, or of Spanish Origin	0.42 (0.38,0.46)	0.44 (0.39,0.49)	0.54 (0.48,0.61)
Multiracial	0.75 (0.66,0.85)	0.74 (0.65,0.86)	0.76 (0.66,0.88)
Native Hawaiian or Pacific Islander	0.24 (0.08,0.77)	0.27 (0.06,1.14)	0.31 (0.07,1.33)
Non-Hispanic White	1 [Reference]	1 [Reference]	1 [Reference]
Other[d]	0.64 (0.55,0.76)	0.74 (0.62,0.90)	0.76 (0.63,0.92)
**Sex**			
Female	1 [Reference]	1 [Reference]	1 [Reference]
Male	0.97 (0.93, 1.01)	0.92 (0.87,0.96)	0.81 (0.77,0.86)
**Sexual Orientation**			
Bisexual	0.72 (0.64,0.83)	0.71 (0.62,0.82)	0.66 (0.56,0.76)
Heterosexual or Straight	1 [Reference]	1 [Reference]	1 [Reference]
Gay or Lesbian	0.96 (0.86,1.08)	0.99 (0.87,1.13)	0.97 (0.85,1.10)
**Childhood family income, $**			
Low Income (<$50,000)	1 [Reference]	1 [Reference]	1 [Reference]
Not Low Income (≥$50,000)	2.08 (1.93,2.24)	1.71 (1.58,1.84)	1.55 (1.43,1.67)
**MCAT Quintile**			
1st Quintile	NA	NA	1 [Reference]
2nd Quintile	NA	NA	1.73 (1.57,1.90)
3rd Quintile	NA	NA	2.41 (2.19,2.65)
4th Quintile	NA	NA	3.02 (2.72,3.35)
5th Quintile	NA	NA	3.61 (3.22,4.04)

Abbreviations: NA, not applicable
[a]Adjusted for clustering by school
[b]Adjusted for clustering by school and for demographic variables (race and ethnicity, sex, sexual orientation, socioeconomic status, childhood family income)
[c]Adjusted for clustering by school and for demographic variables and MCAT quintile
[d] “Other” includes those who responded as Other, Unknown, or Declined to Respond to the Graduation Questionnaire.

eTable3. Odds ratios of membership in Gold Humanism Honor Society by student demographic characteristic with aggregated race/ethnicity

	Odds ratio (95% CI)		
**Characteristic**	(unadjusted) Model[a]	(adjusted) Model[b]	(adjusted) Model[c]
**Race and Ethnicity**			
American Indian or Alaska Native	0.74 (0.43,1.26)	0.87 (0.49,1.53)	0.84 (0.47,1.48)
Asian American	0.83 (0.78,0.89)	0.83 (0.77,0.89)	0.84 (0.78,0.90)
Black or African American	1.50 (1.36,1.65)	1.46 (1.31,1.63)	1.39 (1.24,1.56)
Hispanic, Latino, or of Spanish Origin	0.99 (0.90,1.08)	1.02 (0.92,1.13)	0.99 (0.89,1.09)
Multiracial	0.99 (0.86, 1.13)	0.94 (0.81, 1.09)	0.93 (0.80, 1.09)
Native Hawaiian or Pacific Islander	0.97 (0.44,2.18)	1.07 (0.41,2.82)	1.05 (0.40,2.76)
Non-Hispanic White	1 [Reference]	1 [Reference]	1 [Reference]
Other[d]	1.19 (1.02,1.39)	1.35 (1.13,1.61)	1.34 (1.12,1.60)
**Sex**			
Female	1 [Reference]	1 [Reference]	1 [Reference]
Male	0.63 (0.60,0.66)	0.64 (0.61,0.67)	0.65 (0.62,0.69)
**Sexual Orientation**			
Bisexual	1.28 (1.14,1.45)	1.14 (1.00,1.31)	1.15 (1.01,1.32)
Heterosexual or Straight	1 [Reference]	1 [Reference]	1 [Reference]
Gay or Lesbian	1.19 (1.06,1.34)	1.33 (1.17,1.52)	1.34 (1.17,1.52)
**Childhood family income, $**			
Low Income (<$50,000)	1 [Reference]	1 [Reference]	1 [Reference]
Not Low Income (≥$50,000)	1.09 (1.02,1.17)	1.12 (1.04,1.20)	1.14 (1.06,1.22)
**MCAT Quintile**			
1st Quintile	NA	NA	1 [Reference]
2nd Quintile	NA	NA	0.95 (0.87,1.03)
3rd Quintile	NA	NA	0.95 (0.87,1.04)
4th Quintile	NA	NA	0.89 (0.80,0.98)
5th Quintile	NA	NA	0.81 (0.73,0.91)

Abbreviations: NA, not applicable

[a]Adjusted for clustering by school

[b]Adjusted for clustering by school and for demographic variables (race and ethnicity, sex, sexual orientation, socioeconomic status, childhood family income)

[c]Adjusted for clustering by school and for demographic variables and MCAT quintile

[d] "Other" includes those who responded as Other, Unknown, or Declined to Respond to the Graduation Questionnaire.

© 2026 Yang D et al. *JAMA Network Open*.

eTable4. Odds ratios of membership in Alpha Omega Alpha Honor Society by student demographic characteristic with further disaggregated data

	odds ratio (95% CI)		
**Characteristic**	(unadjusted) Model 1[a]	(adjusted) Model 2[b]	(adjusted) Model 3[c]
**Race and Ethnicity**[d]			
American Indian or Alaska Native	0.58 (0.35,0.96)	0.61 (0.35,1.08)	0.84 (0.47,1.50)
Asian American			
Asian Other[e]	0.55 (0.48,0.64)	0.59 (0.49,0.72)	0.55 (0.46,0.67)
Bangladeshi	0.32 (0.19,0.54)	0.41 (0.23,0.71)	0.36 (0.21,0.62)
Cambodian	0.33 (0.08,1.36)	0.49 (0.11,2.16)	0.53 (0.12,2.34)
Chinese	0.56 (0.49,0.63)	0.60 (0.53,0.69)	0.51 (0.44,0.58)
Filipino	0.44 (0.30,0.63)	0.42 (0.28,0.63)	0.43 (0.29, 0.65)
Indian	0.62 (0.56,0.68)	0.61 (0.55,0.68)	0.56 (0.50,0.63)
Indonesian	0.15 (0.02,1.11)	0.23 (0.03,1.72)	0.24 (0.03,1.83)
Japanese	0.53 (0.34,0.84)	0.50 (0.30,0.84)	0.48 (0.28,0.81)
Korean	0.37 (0.30,0.46)	0.46 (0.37,0.57)	0.41 (0.33,0.51)
Laotian[f]	-	-	-
Pakistani	0.42 (0.32, 0.56)	0.48 (0.35,0.65)	0.46 (0.34,0.63)
Taiwanese	0.46 (0.36,0.59)	0.43 (0.32,0.59)	0.38 (0.28,0.51)
Vietnamese	0.50 (0.40,0.62)	0.58 (0.46,0.73)	0.56 (0.45,0.71)
Black or African American	0.21 (0.18,0.25)	0.25 (0.21,0.30)	0.38 (0.31,0.45)
Hispanic, Latino, or of Spanish Origin	0.42 (0.38,0.46)	0.44 (0.39,0.49)	0.54 (0.48,0.61)
Multiracial	0.75 (0.66,0.85)	0.75 (0.65,0.86)	0.76 (0.66,0.88)
Native Hawaiian or Pacific Islander	0.25 (0.08,0.81)	0.26 (0.06,1.13)	0.31 (0.07,1.31)
Non-Hispanic White	1 [Reference]	1 [Reference]	1 [Reference]
Other[g]	0.64 (0.54,0.76)	0.74 (0.61,0.89)	0.76 (0.63,0.92)
**Sex**			
Female	1 [Reference]	1 [Reference]	1 [Reference]
Male	0.97 (0.93, 1.01)	0.92 (0.87,0.96)	0.81 (0.77,0.86)
**Sexual Orientation**			
Bisexual	0.72 (0.63,0.83)	0.71 (0.61,0.82)	0.66 (0.56,0.76)
Heterosexual or Straight	1 [Reference]	1 [Reference]	1 [Reference]
Gay or Lesbian	0.96 (0.86,1.08)	0.99 (0.87,1.13)	0.96 (0.85,1.10)
**Childhood family income, $**			
Low Income (<$50,000)	1 [Reference]	1 [Reference]	1 [Reference]
Not Low Income (≥$50,000)	2.08 (1.93,2.24)	1.69 (1.57,1.83)	1.14 (1.06,1.22)
**MCAT Quintile**			
1st Quintile	NA	NA	1 [Reference]
2nd Quintile	NA	NA	1.73 (1.58,1.90)
3rd Quintile	NA	NA	2.41 (2.19,2.65)
4th Quintile	NA	NA	3.02 (2.72,3.35)
5th Quintile	NA	NA	3.63 (3.24,4.06)

Abbreviations: NA, not applicable
[a] Adjusted for clustering by school
[b] Adjusted for clustering by school and for demographic variables (race and ethnicity, sex, sexual orientation, socioeconomic status, childhood family income)
[c] Adjusted for clustering by school and for demographic variables and MCAT quintile
[d] Southeast Asian American was further disaggregated into Cambodian, Indonesian, and Laotian
[e] "Asian Other" includes 1) those who identified as Asian but did not provide additional ethnicity and 2) those who identified as multiple Asian ethnicities.
[f] Estimates unavailable due to small sample sizes
[g] "Other" includes those who responded as Other, Unknown, or Declined to Respond to the Graduation Questionnaire.

© 2026 Yang D et al. *JAMA Network Open*.

eTable5. Odds ratio of membership in Gold Humanism Honor Society by student demographic characteristic with further disaggregated data

	Odds ratio (95% CI)		
**Characteristic**	(unadjusted) Model 1[a]	(adjusted) Model 2[b]	(adjusted) Model 3[c]
**Race and Ethnicity**[d]			
American Indian or Alaska Native	0.74 (0.43,1.27)	0.88 (0.49,1.55)	0.84 (0.48,1.50)
Asian American			
Asian Other[e]	0.99 (0.85,1.14)	1.00 (0.83,1.20)	1.00 (0.83,1.21)
Bangladeshi	0.73 (0.46,1.17)	0.68 (0.40,1.18)	0.69 (0.41,1.16)
Cambodian	0.28 (0.04,2.01)	0.31 (0.04,2.38)	0.31 (0.04,2.38)
Chinese	0.65 (0.57,0.75)	0.66 (0.56,0.76)	0.67 (0.58,0.78)
Filipino	0.77 (0.54, 1.10)	0.70 (0.47,1.05)	0.70 (0.47,1.04)
Indian	1.03 (0.92,1.13)	1.04 (0.93,1.16)	1.05 (0.94,1.16)
Indonesian	0.28 (0.04,2.01)	0.35 (0.05,2.61)	0.35 (0.05,2.64)
Japanese	0.99 (0.64,1.51)	0.66 (0.38,1.15)	0.67 (0.38,1.16)
Korean	0.55 (0.44,0.68)	0.54 (0.42,0.68)	0.54 (0.43,0.69)
Laotian[f]	-	-	-
Pakistani	0.84 (0.65,1.09)	0.84 (0.63,1.13)	0.84 (0.63,1.13)
Taiwanese	0.65 (0.50,0.85)	0.66 (0.49,0.90)	0.67 (0.50,0.91)
Vietnamese	0.82 (0.66,1.01)	0.84 (0.67,1.05)	0.84 (0.66,1.05)
Black or African American	1.50 (1.36,1.65)	1.48 (1.32,1.65)	1.41 (1.25,1.59)
Hispanic, Latino, or of Spanish Origin	0.99 (0.91,1.08)	1.02 (0.92,1.13)	0.99 (0.89,1.10)
Multiracial	0.98 (0.86, 1.12)	0.96 (0.85, 1.08)	0.96 (0.85, 1.08)
Native Hawaiian or Pacific Islander	0.91 (0.41,2.04)	1.02 (0.38,2.59)	0.96 (0.37,2.53)
Non-Hispanic White	1 [Reference]	1 [Reference]	1 [Reference]
Other[g]	1.20 (1.02,1.41)	1.36 (1.14,1.63)	1.35 (1.13,1.62)
**Sex**			
Female	1 [Reference]	1 [Reference]	1 [Reference]
Male	0.63 (0.60,0.66)	0.64 (0.60,0.67)	0.65 (0.61,0.69)
**Sexual Orientation**			
Bisexual	1.28 (1.14,1.45)	1.15 (1.00,1.31)	1.16 (1.01,1.32)
Heterosexual or Straight	1 [Reference]	1 [Reference]	1 [Reference]
Gay or Lesbian	1.19 (1.06,1.34)	1.34 (1.17,1.53)	1.34 (1.18,1.53)
**Childhood family income, $**			
Low Income (<$50,000)	1 [Reference]	1 [Reference]	1 [Reference]
Not Low Income (≥$50,000)	1.09 (1.02,1.17)	1.10 (1.02,1.18)	1.12 (1.04,1.20)
**MCAT Quintile**			
1st Quintile	NA	NA	1 [Reference]
2nd Quintile	NA	NA	0.95 (0.88,1.03)
3rd Quintile	NA	NA	0.95 (0.87,1.04)
4th Quintile	NA	NA	0.90 (0.81,0.99)
5th Quintile	NA	NA	0.82 (0.73,0.92)

Abbreviations: NA, not applicable

[a] Adjusted for clustering by school

[b] Adjusted for clustering by school and for demographic variables (race and ethnicity, sex, sexual orientation, socioeconomic status, childhood family income)

[c] Adjusted for clustering by school and for demographic variables and MCAT quintile

[d] Southeast Asian American was further disaggregated into Cambodian, Indonesian, and Laotian

[e] "Asian Other" includes 1) those who identified as Asian but did not provide additional ethnicity and 2) those who identified as multiple Asian ethnicities.

[f] Estimates unavailable due to small sample sizes

[g] "Other" includes those who responded as Other, Unknown, or Declined to Respond to the Graduation Questionnaire.

© 2026 Yang D et al. *JAMA Network Open*.

eTable6. Odds ratios of membership in Alpha Omega Alpha Honor Society by student demographic characteristic, not controlling for medical college admission test

Characteristic	Odds ratio (95% CI) (adjusted) Model[a]
**Race and Ethnicity**	
American Indian or Alaska Native	0.61 (0.35,1.08)
Asian American	
Asian Other[b]	0.59 (0.49,0.72)
Bangladeshi	0.40 (0.23,0.70)
Chinese	0.61 (0.53,0.69)
Filipino	0.42 (0.28,0.63)
Indian	0.61 (0.55,0.68)
Japanese	0.50 (0.30,0.85)
Korean	0.46 (0.37,0.57)
Other Southeast Asian (Cambodian, Indonesian, Laotian)	0.30 (0.09,0.98)
Pakistani	0.48 (0.35,0.65)
Taiwanese	0.43 (0.32,0.58)
Vietnamese	0.58 (0.46,0.73)
Black or African American	0.25 (0.21,0.30)
Hispanic, Latino, or of Spanish Origin	0.44 (0.39,0.49)
Multiracial	0.75 (0.66,0.86)
Native Hawaiian or Pacific Islander	0.25 (0.06,1.05)
Non-Hispanic White	1 [Reference]
Other[c]	0.74 (0.61,0.89)
**Sex**	
Female	1 [Reference]
Male	0.92 (0.87,0.96)
**Sexual Orientation**	
Bisexual	0.71 (0.61,0.81)
Heterosexual or Straight	1 [Reference]
Gay or Lesbian	0.99 (0.87,1.13)
**Childhood family income, $**	
Low Income (<$50,000)	1 [Reference]
Not Low Income (≥$50,000)	1.69 (1.57,1.83)
**MCAT Quintile**	
1st Quintile	NA
2nd Quintile	NA
3rd Quintile	NA
4th Quintile	NA
5th Quintile	NA

Abbreviations: NA, not applicable

[a] Adjusted for clustering by school and for demographic variables (race and ethnicity, sex, sexual orientation, socioeconomic status, childhood family income)

[b] "Asian Other" includes 1) those who identified as Asian but did not provide additional ethnicity and 2) those who identified as multiple Asian ethnicities.

[c] "Other" includes those who responded as Other, Unknown, or Declined to Respond to the Graduation Questionnaire.

© 2026 Yang D et al. *JAMA Network Open*.

eTable7. Odds ratios of membership in Gold Humanism Honor Society by student demographic characteristic, not controlling for medical college admission test

Characteristic	Odds ratio (95% CI) (adjusted) Model[a]
**Race and Ethnicity**	
American Indian or Alaska Native	0.87 (0.49,1.53)
Asian American	
Asian Other[b]	1.00 (0.83,1.20)
Bangladeshi	0.68 (0.40,1.14)
Chinese	0.66 (0.56,0.76)
Filipino	0.70 (0.47,1.05)
Indian	1.04 (0.93,1.15)
Japanese	0.66 (0.38,1.15)
Korean	0.54 (0.42,0.69)
Other Southeast Asian (Cambodian, Indonesian, Laotian)	0.30 (0.07,1.24)
Pakistani	0.84 (0.63,1.12)
Taiwanese	0.66 (0.48,0.90)
Vietnamese	0.84 (0.67,1.05)
Black or African American	1.45 (1.30,1.62)
Hispanic, Latino, or of Spanish Origin	1.02 (0.92,1.12)
Multiracial	0.93 (0.80,1.09)
Native Hawaiian or Pacific Islander	1.01 (0.38,2.66)
Non-Hispanic White	1 [Reference]
Other[c]	1.35 (1.12,1.61)
**Sex**	
Female	1 [Reference]
Male	0.64 (0.60,0.67)
**Sexual Orientation**	
Bisexual	1.15 (1.00,1.31)
Heterosexual or Straight	1 [Reference]
Gay or Lesbian	1.34 (1.17,1.53)
**Childhood family income, $**	
Low Income (<$50,000)	1 [Reference]
Not Low Income (≥$50,000)	1.10 (1.02,1.18)
**MCAT Quintile**	
1st Quintile	NA
2nd Quintile	NA
3rd Quintile	NA
4th Quintile	NA
5th Quintile	NA

Abbreviations: NA, not applicable

[a] Adjusted for clustering by school and for demographic variables (race and ethnicity, sex, sexual orientation, socioeconomic status, childhood family income)

[b] "Asian Other" includes 1) those who identified as Asian but did not provide additional ethnicity and 2) those who identified as multiple Asian ethnicities.

[c] "Other" includes those who responded as Other, Unknown, or Declined to Respond to the Graduation Questionnaire.

© 2026 Yang D et al. *JAMA Network Open*.
