# Supplementary material for: Disparities for Asian American Medical Students in Alpha Omega Alpha and Gold Humanism Honor Societies
Source: JAMA Netw Open. 2026 Apr 6;9(4):e265168. doi: 10.1001/jamanetworkopen.2026.5168 (PMC13054621; doi:10.1001/jamanetworkopen.2026.5168)
Supplement: Supplement 2. — Data Sharing Statement [file jamanetwopen-e265168-s002.pdf]

Data Sharing Statement

Yang. Disparities for Asian American Medical Students in Alpha Omega Alpha and Gold Humanism Honor Societies. *JAMA Netw Open*. Published April 06, 2026.
doi:10.1001/jamanetworkopen.2026.5168

Data
Data available: No

Additional Information
Explanation for why data not available: This data is made available through the AAMC
